# Inertial Sensor Measurements of Upper-Limb Kinematics in Stroke Patients in Clinic and Home Environment

**DOI:** 10.3389/fbioe.2018.00027

**Published:** 2018-04-12

**Authors:** Jeremia P. O. Held, Bart Klaassen, Albert Eenhoorn, Bert-Jan F. van Beijnum, Jaap H. Buurke, Peter H. Veltink, Andreas R. Luft

**Affiliations:** ^1^Division of Vascular Neurology and Neurorehabilitation, Department of Neurology, University Hospital of Zurich, Zurich, Switzerland; ^2^Biomedical Signals and Systems, MIRA—Institute for Biomedical Technology and Technical Medicine, University of Twente, Enschede, Netherlands; ^3^cereneo, Center for Neurology and Rehabilitation, Vitznau, Switzerland; ^4^Faculty of Behavioural, Management and Social Sciences, University of Twente, Enschede, Netherlands; ^5^Roessingh Research and Development B.V., Enschede, Netherlands

**Keywords:** rehabilitation, sensors, stroke, daily-life activities, monitoring, assessments, kinematic

## Abstract

**Background:**

Upper-limb impairments in stroke patients are usually measured in clinical setting using standard clinical assessment. In addition, kinematic analysis using opto-electronic systems has been used in the laboratory setting to map arm recovery. Such kinematic measurements cannot capture the actual function of the upper extremity in daily life. The aim of this study is to longitudinally explore the complementarity of post-stroke upper-limb recovery measured by standard clinical assessments and daily-life recorded kinematics.

**Methods:**

The study was designed as an observational, single-group study to evaluate rehabilitation progress in a clinical and home environment, with a full-body sensor system in stroke patients. Kinematic data were recorded with a full-body motion capture suit during clinical assessment and self-directed activities of daily living. The measurements were performed at three time points for 3 h: (1) 2 weeks before discharge of the rehabilitation clinic, (2) right after discharge, and (3) 4 weeks after discharge. The kinematic analysis of reaching movements uses the position and orientation of each body segment to derive the joint angles. Newly developed metrics for classifying activity and quality of upper extremity movement were applied.

**Results:**

The data of four stroke patients (three mildly impaired, one sever impaired) were included in this study. The arm motor function assessment improved during the inpatient rehabilitation, but declined in the first 4 weeks after discharge. A change in the data (kinematics and new metrics) from the daily-life recording was seen in in all patients. Despite this worsening patients increased the number of reaches they performed during daily life in their home environment.

**Conclusion:**

It is feasible to measure arm kinematics using Inertial Measurement Unit sensors during daily life in stroke patients at the different stages of rehabilitation. Our results from the daily-life recordings complemented the data from the clinical assessments and illustrate the potential to identify stroke patient characteristics, based on kinematics, reaching counts, and work area.

**Clinical Trial Registration:**

https://clinicaltrials.gov, identifier NCT02118363.

## Introduction

Stroke is the second most common cause of disability worldwide (Murray et al., 2012). After stroke, approximately 50% of all patients have long-term impairments of upper-limb motor function (Kwakkel et al., 2003). These impairments and activities are usually measured in the laboratory with standard clinical assessments such as the Fugl-Meyer Assessment—Upper Extremity subscale (FMA-UE) (Fugl-Meyer et al., 1975) and the Action Research Arm Test (ARAT) (Lyle, 1981). In the past decade, kinematic analysis of the upper extremity using opto-electronic systems in a clinical setting (Levin, 1996; Cirstea and Levin, 2000; Alt Murphy et al., 2011, 2013; Subramanian and Levin, 2011), has been applied as well to evaluate upper-limb motor recovery after stroke (de los Reyes-Guzman et al., 2014). However, these clinical assessments reflect the patients’ best abilities as they are encouraged by an assessor. This test situation does not reflect daily-life upper-limb use (Stewart and Cramer, 2013).

In stroke clinical trials, acceleration sensors have been used to measure the patient arm-activities in real world (Uswatte et al., 2005). Although accelerometer sensors can be used to measure movements in the sagittal plane (Leuenberger et al., 2016), they cannot provide information regarding three-dimensional (3D) movements of the upper limb. To measure movement quality kinematic metrics from optical motion capture systems quantify the patients’ motor abilities on a body function level but remain restricted to a motion capture laboratory and cannot be used in daily life. New technologies such as wearable inertial measurement units (IMUs) make it possible to quantify upper-limb motor function in daily life (Patel et al., 2012; Steins et al., 2014; van Meulen et al., 2015). IMUs are able to measure movement kinematics without being restricted to certain location (Roetenberg et al., 2009). The application of IMUs in a laboratory setting has been compared with standard clinical assessments and showed a good correlation to clinical assessments (e.g., FMA-UE) and short simulated daily-life tasks (van Meulen et al., 2015). This study indicated that achievements during rehabilitation are incompletely implemented in daily life (van Meulen et al., 2016).

New technologies, with the possibility to continuously perform daily-life monitoring of functional activities in real life, can monitor response to a new therapy, guide recovery (Schweighofer et al., 2009), and may be valuable tools to measure outcomes in clinical trials. For patients who need continuing training after inpatient rehabilitation, it is important to monitor progress and deterioration.

So far it was not possible to study upper-limb motor recovery during daily life in terms of kinematics at different stages after inpatient stroke rehabilitation. The development of new sensor technology made it possible to detect movement kinematics in stroke patients (van Meulen et al., 2016).

### Aim of the Study

The aim is to longitudinally explore the complementary between post-stroke upper-limb recovery measured with standard clinical assessments and daily-life kinematic recordings using IMUs during the transition from inpatient rehabilitation to home. These data could be valuable in planning and monitoring outpatient rehabilitation therapy (Uswatte et al., 2000; Andre et al., 2004).

## Materials and Methods

### Study Design

**Figure 1:**
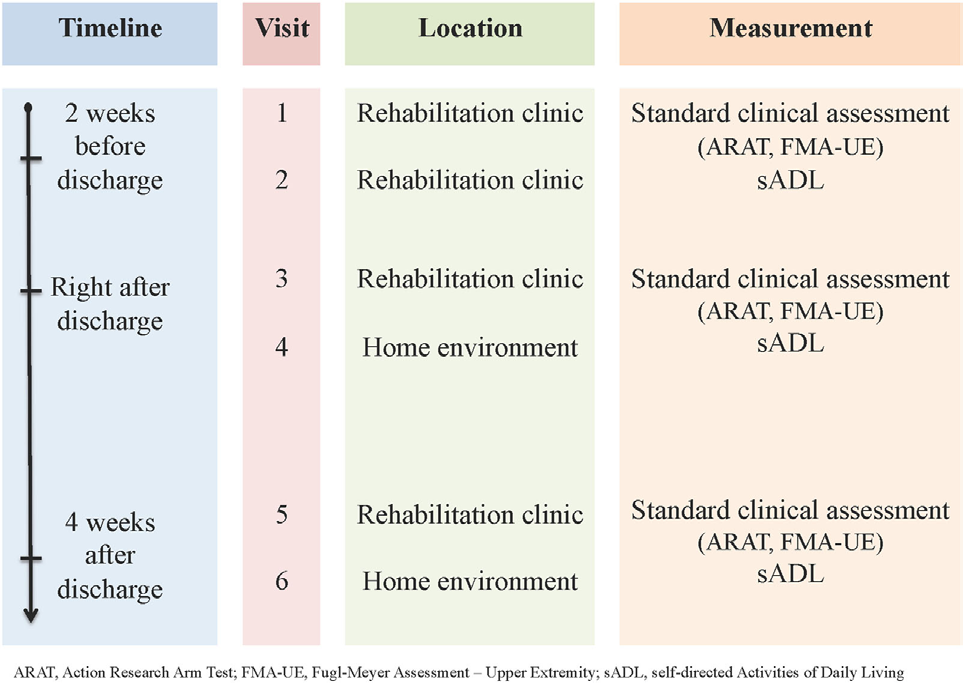
Overview of visits and assessments. ARAT, Action Research Arm Test; FMA-UE, Fugl-Meyer Assessment––Upper Extremity; sADL, self-directed Activities of Daily Living.

The study was designed as an observational, single-group study to evaluate rehabilitation progress (over 6 weeks) in a clinical and home environment, with a full-body IMU system in stroke patients (Figure 1). Stroke subjects with a first-ever ischemic stroke were admitted to cereneo—Center for Neurology and Rehabilitation, Vitznau, Switzerland. Inclusion criteria were (1) age between 35 and 80 years of age, (2) a hemiparesis as a result of a single unilateral stroke, (3) able to lift their effected arm against gravity, and (4) to walk 10 m without supervision. Exclusion criteria were the inability to understand questionnaires and inability to perform given instructions. Patients were recruited between January 2014 and January 2015.

The study was approved by the Cantonal Ethics Committee Northwest and Central Switzerland (EKNZ 13101). All subjects gave written informed consent in accordance with the declaration of Helsinki.

### Measurement System

Kinematic data were recorded with an Xsens full-body motion capture suit. Each IMU consists of a 3D accelerometer, a 3D magnetometer, and a 3D gyroscope (Xsens Technologies, Enschede, Netherlands). To measure full-body kinematics, 14 IMUs were positioned by a therapist on the following body segments: on the instep of both feet, lower legs (medial of the tuberosity tibia), upper legs (middle part of the upper leg, on the Iliotibial tract), lower arms (3-cm distal of the wrist), upper arms (15-cm distal from the acromion), both shoulders (spine of the scapula), sternum, and the sacrum (Klaassen et al., 2015). Data of all sensors were captured in Xsens MVN Studio software to estimate full-body 3D kinematics, e.g., each body segment orientation, relative segment position and joint angles (Roetenberg et al., 2005), with a sampling rate of 20 Hz. This frequency was found to be adequate for the developed daily-life movement metrics as internal sensor data were captured at a higher frequency (Klaassen et al., 2015; van Meulen et al., 2016).

Data were transferred wirelessly to a base station (Awinda Station, Xsens, the Netherlands), and connected to a laptop *via* USB. The base station allowed a maximal range of 10 m to the stroke patients. A trained research therapist monitored the system for sensor loss or system failure. To ensure good sensor quality data, the calibration procedure was performed during the measurement, if the patient changed floor level or when changes in the movement reconstructions where found indicated a sensor drift. The therapist never encouraged the patient to perform any activity. If the patient was out of range a therapist took the laptop and the base station after to the patient.

### Measurements

The measurements with the full-body IMU system have been performed during the standard clinical assessment and during of self-directed activities of daily living (sADL). Clinical assessments included arm motor function assessment using the FMA-UE (Fugl-Meyer et al., 1975) and the ARAT (Lyle, 1981) to assess the patients’ arm activities. In addition, the Test of Attentional Performance was included, to test the existence of a neglect (Zimmermann and Fimm, 2002). The assessments were performed in the clinic by a trained therapist. The sADLs were performed in the patient leisure time (clinic) and in house without any instructions. sADL data at each time points were collected for 3 h. Measuring stroke patients’ sADL that could not be possible to performed while wearing the full-body IMU system (dressing, go to the restroom showering) were excluded from the daily-life measurements. Data were continuously recorded during sADL. To ensure manageable file sizes, data were saved every 10–15 min, after which recordings were continued without influencing the patient daily-life activities.

Measurements were performed at three time points for 3 h (Figure 1): (1) 2 weeks before discharge of the rehabilitation clinic, (2) right after discharge, and (3) 4 weeks after discharge.

### Sensor Data

The Xsens MVN studio software (MVN Studio, Xsens, the Netherlands) was used for data capturing. Each body segment position and orientation was estimated using a Kalman filter (Xsens Kalman Filter, XKF) included in the software to generate a 3D reconstruction (Roetenberg et al., 2009). Measurement reports, including new metrics for stroke patient evaluation, were generated in an offline environment using MATLAB^®^ (The MathWorks Inc., Natick, MA, USA). The measurement reports use the position and orientation of each body segment to derive the joint angles. The accuracy was approximately 5 mm for position and 3° for orientation measurements of the system for each body segment (Roetenberg et al., 2007).

Previously developed metrics for classifying activities and assessing the quality of lower and upper extremity movements were applied (van Meulen et al., 2016). Classification of the activities included posture detection (sitting or standing), walking detection, arm movements, and reaching detection of the affected and non-affected arm. To present large amount of aggregated sADL data in a consistent way, descriptive statistics, including average joint range of motion (RoM) (from min to max) during a reaching movement and SDs was used (van Meulen et al., 2016).

For the upper extremities (affected and non-affected arm), the elbow and shoulder RoM, the hand position relative to the pelvis in the transversal plane, the maximum reaching distance and the reaching counts were calculated. Reaching counts were based on a hand displacement of more than 10 cm away from the preferred hand position (the average hand position relative to the pelvis) (van Meulen et al., 2016). Based on this metric, the ratio of reaching counts between non-impaired and the impaired side was calculated. The reaching distance was estimated by evaluating consecutive positions of each hand expressed in the pelvis and the sternum coordinate system (Steins et al., 2014). Based on these data, the distribution of the patient’s hand position in the horizontal plane was visualized. The usability of these metrics for the objective evaluation of motor performance Stroke patients were found to be adequate, while a combination of metrics provided better insight in the patient sADL performance (Klaassen et al., 2017).

## Results

### Subjects Baseline Characteristics

Eight stroke patients (48–55 years of age) were included in this study. They had an inpatient rehabilitation stay of at least 1 month. There was a full longitudinal data set available for four of eight patients (Table 1). Due to technical problems related to sensor data loss and sensor drift, the other patients could not be included in the analysis.

**Table 1:** Baseline characteristics of four stroke patients.

	P1	P2	P3	P4
Time post-stroke (months)	12	1	4	4
Affected side	Left	Left	Right	Right
Dominant side	Right	Right	Right	Right
Neglect Test (TAP^a^)	None	7 left	None	None
FMA-UE^b^ (Total)	57	55	57	7
FMA-UE (proximal)	30	31	31	7
FMA-UE (hand/wrist)	23	20	21	0
FMA-UE (coordination)	4	4	5	0
ARAT^c^ (total)	57	52	57	3
ARAT (grasp)	18	18	18	3
ARAT (grip)	12	11	12	0
ARAT (pinch)	18	14	18	0
ARAT (gross movement)	9	9	9	0

*^a^Test of Attentional Performance––Subtest Visual Field (Absence on one side)*.

*^b^Fugl–Meyer Assessment––Upper Extremity (0–66 points)*.

*^c^Action Research Arm Test (0–57 points)*.

### Standard Clinical Assessments

**Figure 2:**
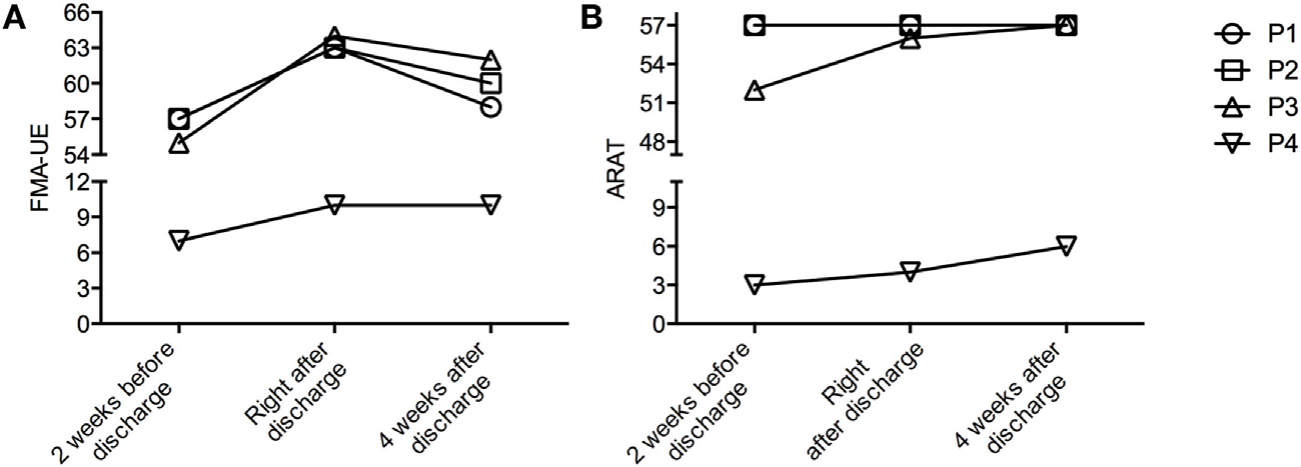
Change in clinical assessment at the three different time points. **(A)** Fugl-Meyer Assessment––Upper Extremity (FMA-UE)––maximum 66 points. **(B)** Action Research Arm Test (ARAT)––maximum 57 points.

Three patients (P1, P2, and P3) had mild motor upper-limb impairments (FMA-UE ≥ 53/66 points) and one (P4) had severe motor impairment of the upper extremity (7/66 points). The arm motor function assessment (FMA-UE) improved seven points in the three patients (P1, P2, P3) with a high FMA-UE from baseline to right after discharge, but declined 4 weeks after discharge (Figure 2A). In the ARAT two patients (P3 and P4) improved slightly in arm activities (Figure 2B). One patient was diagnosed with a neglect (P2) patient, which improved over time from 7 to 4 omissions in the Test of Attentional Performance.

### Continuous Measurement of sADL

**Figure 3:**
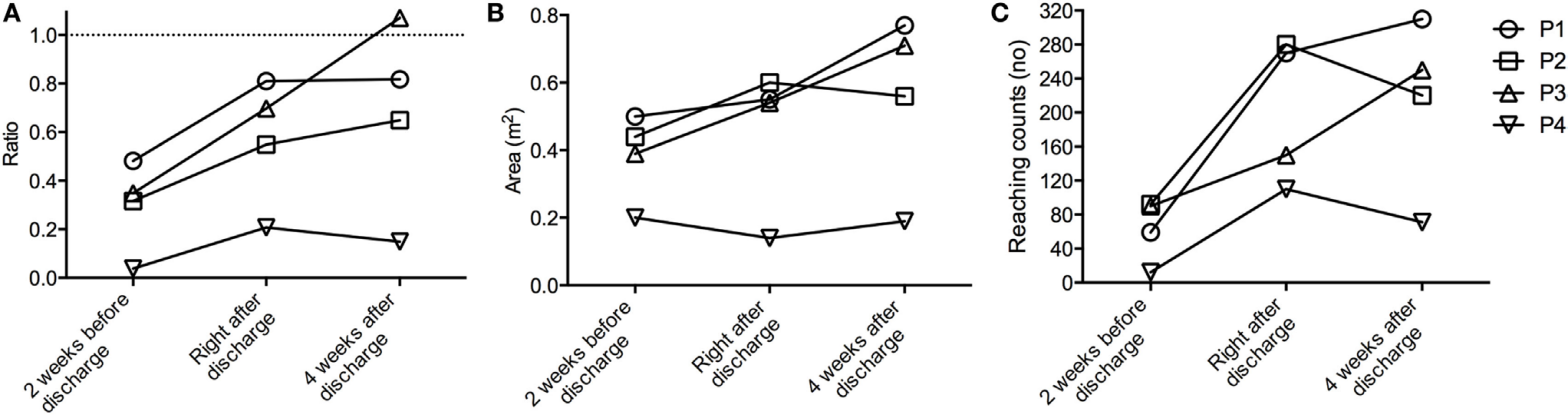
Self-directed Activities of Daily Living (sADL). **(A)** Ratio of reaching counts between non-impaired and the impaired side. **(B)** Reaching area of the impaired side in the different stages of the rehabilitation. **(C)** Reaching counts of the affected side for all patients during sADL, measured over time 3 h.

**Figure 4:**
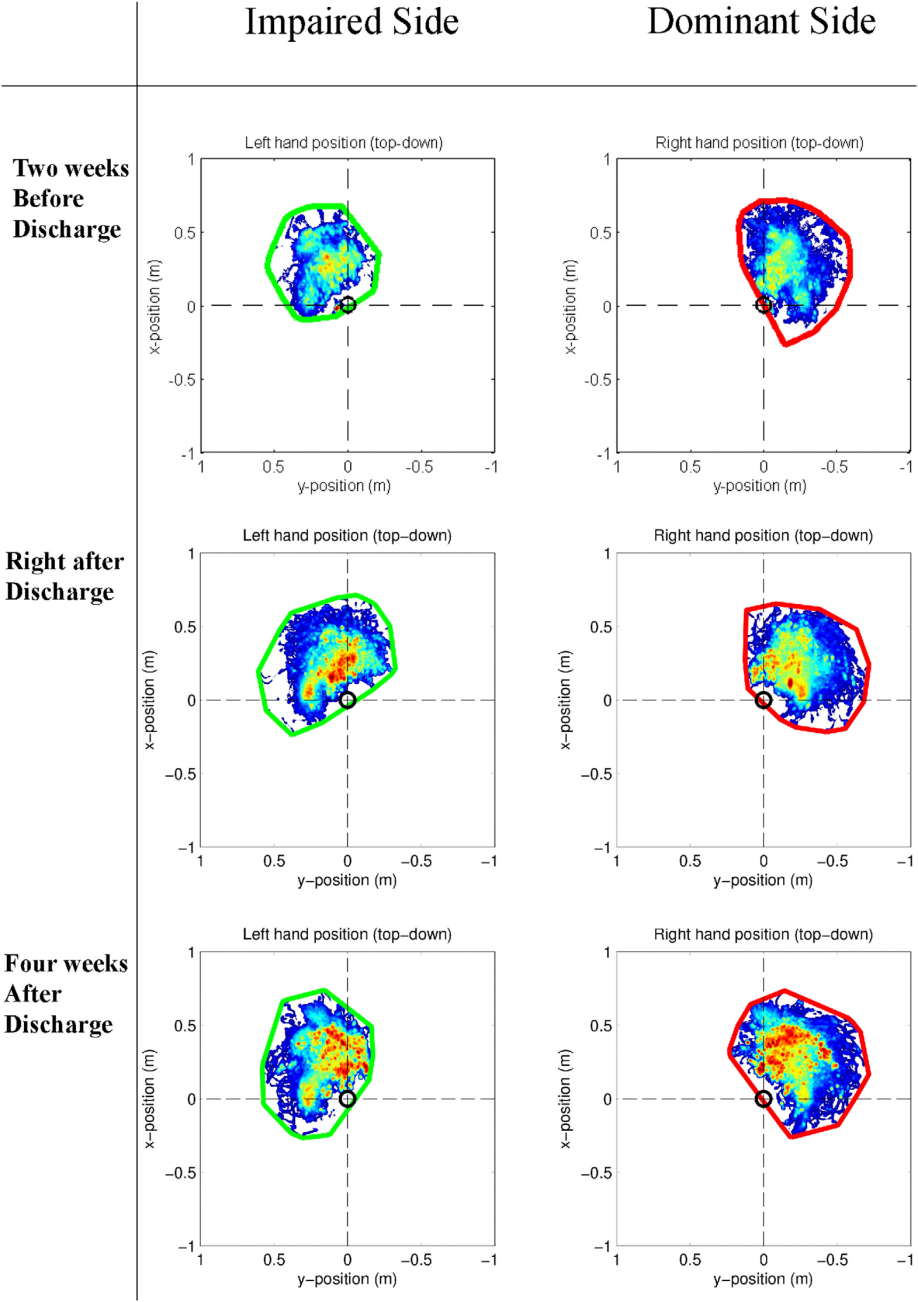
Example of the distribution of the hand position relative to the pelvis in the horizontal plane (colors indicate the total time during the selected time slot at which the hand is in a certain position, where a darker color reflects a longer time) of P2 at the three different stages in the rehabilitation process during self-directed activities of daily living. The encircled trajectory (left hand = green, right hand = red) determines the reaching area of the patient.

Table 2 shows the kinematic parameters collected during reaching movements: elbow flexion, shoulder abduction, and shoulder flexion (mean ± SD over all reaching movements). The patient with the most severe motor impairments (P4) had low shoulder abduction angles at all time points and after discharge high values of elbow flexion. P2 showed improvements in all kinematic data and kept them at least partially (even further improved in shoulder flexion). The kinematic data for the other patients (P1, P3) did not show relevant over the course of rehabilitation. A change in the new metrics (reaching counts, reaching area, workspace) was seen in all subjects. Reaching counts on the impaired side from average 63 reaches (in the clinic) to 202 reaches after discharge (Figure 3C). Also the ratio of the reaching counts between the non-impaired and the impaired side increases 26.8% (Figure 3A). Mildly affected stroke patients (P1, P2, P3) increased the reaching area, measured during self-directed daily activities after discharge (Figures 3B and 4; Figures S1–S3 in Supplementary Material). Furthermore, P3 (right affected/right handed) could persist the trend of increasing the reaching area (0.17 m^2^) and reaching counts (37.3%). This is in contrast to P2 (right handed/left affected), who slightly decrease his workspace after discharge (0.03 m^2^) and showed a slow increased in the reaching counts (12%) 4 weeks after discharge. Additionally, it appears that P2 crosses the midline less with the right non-impaired hand as, compared with the impaired hand. The impaired hand is neglecting the non-impaired side (Figure 4).

**Table 2:** Kinematic data during a reaching movements (average joint range of motion and SD) of the effected side, for all patients (P1, P2, P3, and P4) during self-directed activities of daily living, measured over time 3 h.

Parameter	Time point	P1		P2		P3		P4	
		Average	SD	Average	SD	Average	SD	Average	SD
Elbow Flexion (°)	2 weeks before discharge	26.70	25.00	10.3	14	17.3	14	20.4	19
	Right after discharge	25.20	22.00	19.1	18	19.7	21	42.9	64
	4 weeks after discharge	29.20	35.00	14.7	14	19.1	22	21.8	25
Shoulder Abduction (°)	2 weeks before discharge	11.40	7.10	6.25	7.6	10	9	3.7	5.4
	Right after discharge	11.60	9.60	11.1	10	12	13	5.8	4.3
	4 weeks after discharge	12.80	11	10.1	11	12	12	5.1	4.4
Shoulder Flexion (°)	2 weeks before discharge	14.3	14	23.4	73	39	88	93	160
	Right after discharge	21.3	19	65.9	130	100	150	89	140
	4 weeks after discharge	18.1	16	122	160	83	140	36	81

## Discussion

These results demonstrate the feasibility of the method to measure upper-limb kinematics, with an IMU-based motion capture system at different stages of stroke rehabilitation and during sADL and the concordance to standard clinical assessment. Although this study did not aim to compare the clinical data with the kinematic measurements, we observed a difference between the clinical assessments and the sADL measures, not only in a cross-sectional manner but also over time. The proposed metrics (reaching count, area, workspace) provide additional information as it shows an evolution, while standard clinical assessments remained stable over time after discharge. This present explorative study shows that patients with high arm function (FMA-UE) can change clinically relevant in rehabilitation (Page et al., 2012). The data from the sADL measurements including the metrics from the sensors and the standard clinical test made it possible to characterize patients during daily life (participation level) (Uswatte et al., 2000; Andre et al., 2004). An understanding of the discrepancy between the clinical assessments, where the patient is encouraged by the therapist, and the patients’ performance at home would help to develop tailored, innovative rehabilitation interventions, which target engagement of upper-limb use in daily life. According to the current literature, this is the first study that analyzed kinematic data measured outside the clinic environment at different stages of stroke rehabilitation. While performing daily-life activities a change in arm kinematics after in-patient rehabilitation could be observed.

For the mildly impaired subjects, this was observable in the metrics reaching area, reaching counts, and ratio of reaching counts (Figure 3), but not in the shoulder and elbow angle ranges (Table 2). In the severely impaired patient, no change in the shoulder abduction angles and no change in the working area were found. This could be caused by the weakness of elbow extensors under higher shoulder load (abduction angles), which also contribute to reductions in work area (Sukal et al., 2007).

Previous studies using accelerometer data to calculate the ratio of impaired and non-impaired upper-limb use reported a less-symmetric and less-intense real-world bilateral upper-limb activity compared with healthy subjects (van der Pas et al., 2011; Michielsen et al., 2012; Bailey et al., 2015). Our findings are supplemented by the low amount of reaching counts on the impaired side and the difference in hand position, found in our current study that indicate a reduction of real-world upper-limb use even in mildly effected stroke patients. Also, the differences between people living in the community and inpatient rehabilitation have not been reported in previous studies (Bailey et al., 2015). Furthermore, the increase in reaching counts ratio between the impaired and non-impaired arm after rehabilitation in all patients would also suggest that patients have to be motivated to use their hands more in the leisure time during the inpatient stay.

When looking at the single arm use (Figure 4), the new developed metric (work area) offers the possibility to assess and plan interventions for motor neglect. These results supports the findings from Ogourtsova, Archambault (Ogourtsova et al., 2015) that neglect contribute to deficits observed in action execution of the non-affected limb.

### Limitation

To measure stroke patients, sADLs are challenging but promising. The main limitation of this feasibility study is the low number of stroke patients included. From eight post-stroke patients who where equipped with the full-body motion capture system, data from only four patients were suitable for analysis. The data from the four excluded patients were not usable due to sensor orientation (sensor drift and sensor placement) and transmitting problems from sensors to the receiving device. The importance of the sensor calibration procedures, the influence of the environmental factors (e.g., change in floor levels, electronic devices in home), the duration of measurements, and the complexity of activities of the patients affected the measurements (Robert-Lachaine et al., 2017). This could be solved with more robust sensing and communication systems in the future. It is unclear what patients did during the 3 h of sADL, as tasks could highly influence upper-limb kinematics.

A combination of sensors and a more extensive activity monitoring system including a markerless camera system could increase the knowledge about the patient performance (Sevrin et al., 2016). Also the obtrusive measurement setup (14 sensors) makes it less suitable for long-term measurements, without technical support in stroke subjects. Furthermore, the presence of the therapist could influence the patient performance during the measurement. A reduced sensor set would improve the problem of obtrusiveness (Leuenberger et al., 2016; van Meulen et al., 2017).

Moreover, a group analysis was not possible because of data loss of four subjects and the heterogeneity of the stroke population.

## Conclusion

This study showed the feasibility of measuring kinematics in stroke patients at the different stages of rehabilitation. Our results illustrate that certain metrics derived from kinematic data are likely more sensitive to changes as compared with clinical assessments. Measuring with a full-body IMU system allows a quantification of movement quality outside a laboratory environment. Future studies are needed to optimize the technology, better characterize the metrics derived from IMUs, and include more post-stroke patients to profile the rehabilitation process.

## Ethics Statement

The study was approved by the Cantonal Ethics Committee Northwest and Central Switzerland (EKNZ 13101). All subjects gave written informed consent in accordance with the declaration of Helsinki.

## Author Contributions

JH drafted the manuscript. JH and AE provided the data of the stroke subjects. JH, BK, JB, AL, B-JB, and PV assisted with data interpretation, and helped to draft the manuscript. AL and PV supervised the research. All authors read and approved the final manuscript.

## Conflict of Interest Statement

The study sponsor Prof. Dr. AL is scientific advisor for Hocoma AG (Volketswil, Switzerland), which develops rehabilitation technology. However, there is no link between Hocoma and Xsens. With that, all authors declare that the research was conducted in the absence of any commercial or financial relationships that could be construed as a potential conflict of interest.

## Supplementary Material

Figure S1 The distribution of the hand position relative to the pelvis (colors indicate the total time during the selected time slot at which the hand is in a certain position: dark red = most frequent position, blue = least-frequent position) of P1 at the three different stages in the rehabilitation process during self-directed activities of daily living. The encircled trajectory (left hand = green, right hand = red) determine the reaching area of the patient.

Figure S2 The distribution of the hand position relative to the pelvis (colors indicate the total time during the selected time slot at which the hand is in a certain position: dark red = most frequent position, blue = least-frequent position) of P3 at the three different stages in the rehabilitation process during self-directed activities of daily living. The encircled trajectory (left hand = green, right hand = red) determine the reaching area of the patient.

Figure S3 The distribution of the hand position relative to the pelvis (colors indicate the total time during the selected time slot at which the hand is in a certain position: dark red = most frequent position, blue = least-frequent position) of P4 at the three different stages in the rehabilitation process during self-directed activities of daily living. The encircled trajectory (left hand = green, right hand = red) determine the reaching area of the patient.

## Abbreviations

ARAT, Action Research Arm Test; FMA-UE, Fugl-Meyer assessment—upper extremity; IMU, inertial measurement unit; RoM, range of motion; sADL, self-directed activities of daily living.
