# Supplementary figures and images for: Inertial Sensor Measurements of Upper-Limb Kinematics in Stroke Patients in Clinic and Home Environment
Source: Front Bioeng Biotechnol. 2018 Apr 12;6:27. doi: 10.3389/fbioe.2018.00027 (PMC5906540; doi:10.3389/fbioe.2018.00027)

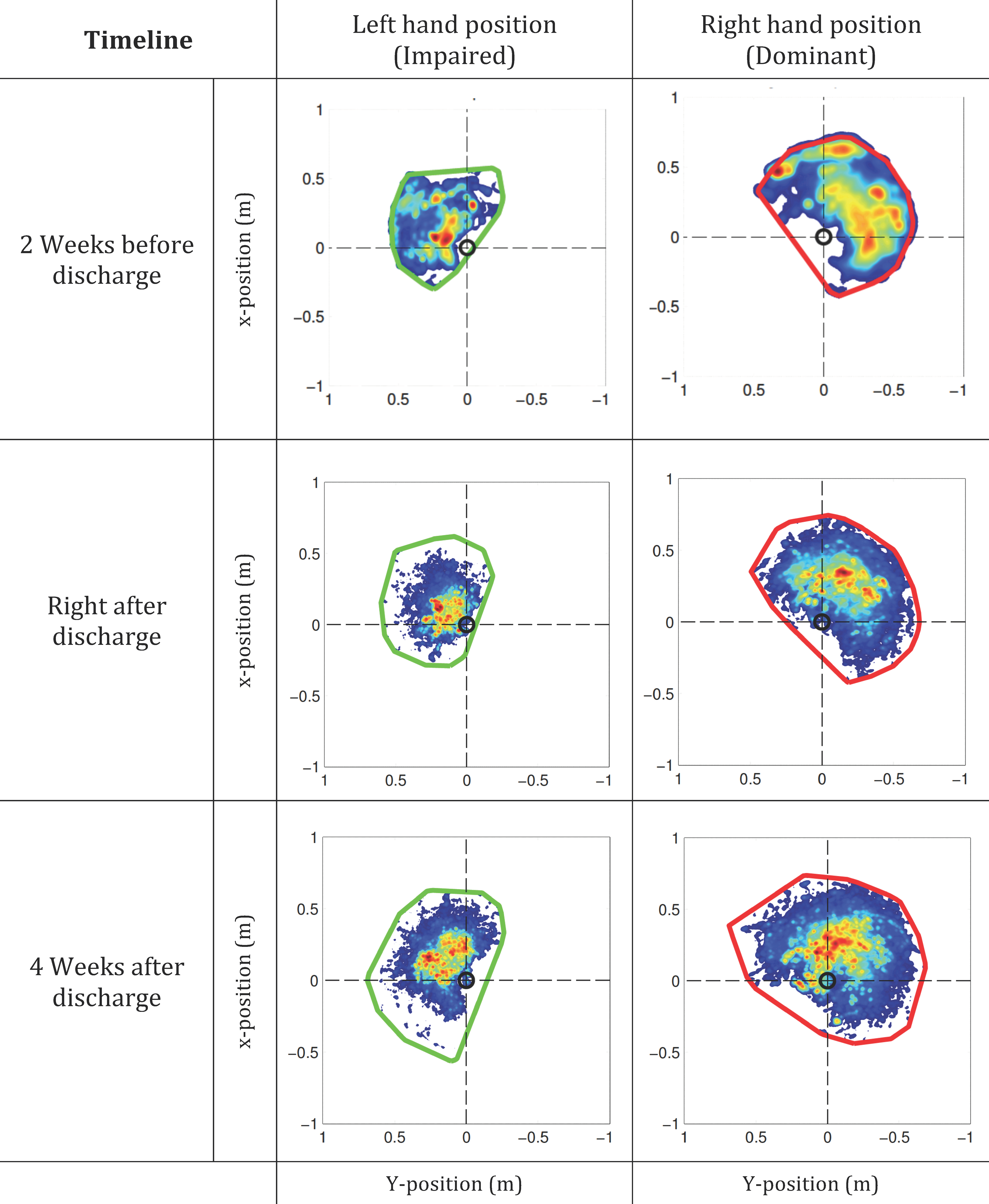

Supplement: Figure S1 — The distribution of the hand position relative to the pelvis (colors indicate the total time during the selected time slot at which the hand is in a certain position: dark red = most frequent position, blue = least-frequent position) of P1 at the three different stages in the rehabilitation process during self-directed activities of daily living. The encircled trajectory (left hand = green, right hand = red) determine the reaching area of the patient. [file image_1.tif]

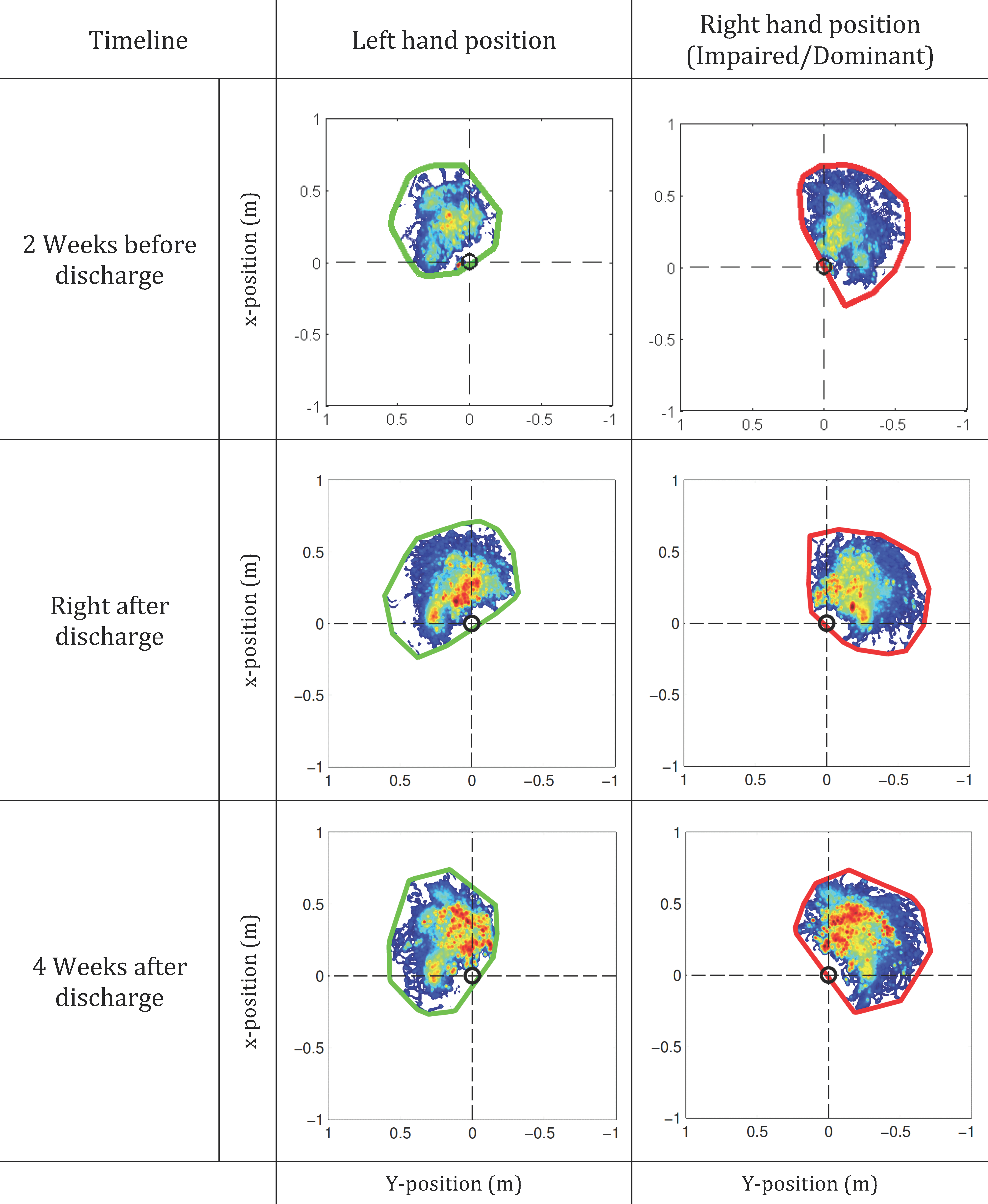

Supplement: Figure S2 — The distribution of the hand position relative to the pelvis (colors indicate the total time during the selected time slot at which the hand is in a certain position: dark red = most frequent position, blue = least-frequent position) of P3 at the three different stages in the rehabilitation process during self-directed activities of daily living. The encircled trajectory (left hand = green, right hand = red) determine the reaching area of the patient. [file image_2.tif]

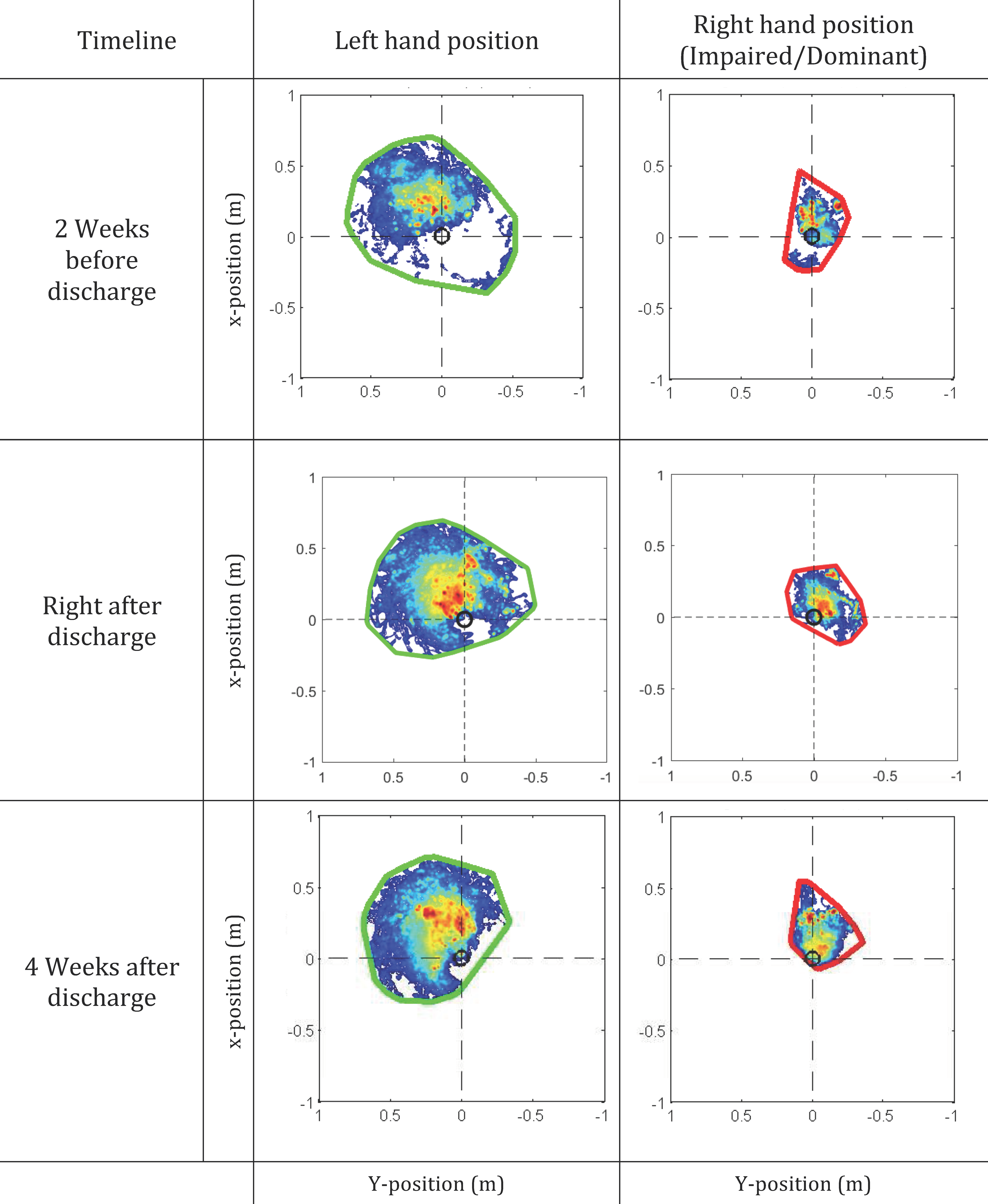

Supplement: Figure S3 — The distribution of the hand position relative to the pelvis (colors indicate the total time during the selected time slot at which the hand is in a certain position: dark red = most frequent position, blue = least-frequent position) of P4 at the three different stages in the rehabilitation process during self-directed activities of daily living. The encircled trajectory (left hand = green, right hand = red) determine the reaching area of the patient. [file image_3.tif]
